# Using Omics to Study Leprosy, Tuberculosis, and Other Mycobacterial Diseases

**DOI:** 10.3389/fcimb.2022.792617

**Published:** 2022-02-24

**Authors:** Naseem Ahamad, Saurabh Gupta, Deepak Parashar

**Affiliations:** ^1^ Department of Oral and Maxillofacial Diagnostic Sciences, College of Dentistry, University of Florida, Gainesville, FL, United States; ^2^ Department of Biotechnology, GLA University, Mathura, India; ^3^ Department of Obstetrics and Gynecology, Medical College of Wisconsin, Milwaukee, WI, United States

**Keywords:** tuberculosis, leprosy, mycobacteria, genomics, transcriptomics, proteomics, metabolomics, lipidomics

## Abstract

Mycobacteria are members of the Actinomycetales order, and they are classified into one family, Mycobacteriaceae. More than 20 mycobacterial species cause disease in humans. The Mycobacterium group, called the *Mycobacterium tuberculosis* complex (MTBC), has nine closely related species that cause tuberculosis in animals and humans. TB can be detected worldwide and one-fourth of the world’s population is contaminated with tuberculosis. According to the WHO, about two million dies from it, and more than nine million people are newly infected with TB each year. *Mycobacterium tuberculosis* (*M. tuberculosis*) is the most potential causative agent of tuberculosis and prompts enormous mortality and morbidity worldwide due to the incompletely understood pathogenesis of human tuberculosis. Moreover, modern diagnostic approaches for human tuberculosis are inefficient and have many lacks, while MTBC species can modulate host immune response and escape host immune attacks to sustain in the human body. “Multi-omics” strategies such as genomics, transcriptomics, proteomics, metabolomics, and deep sequencing technologies could be a comprehensive strategy to investigate the pathogenesis of mycobacterial species in humans and offer significant discovery to find out biomarkers at the early stage of disease in the host. Thus, in this review, we attempt to understand an overview of the mission of “omics” approaches in mycobacterial pathogenesis, including tuberculosis, leprosy, and other mycobacterial diseases.

## Introduction

Mycobacterial disease such as tuberculosis (TB) continues to be one of the world’s leading infectious diseases, claiming over 1.5 million lives annually or 4000 lives each day, and the Global TB Report 2020 predicts that 10 million new cases and 1.4 million fatalities occurred in 2019 (Chakaya et al., 2021; Parvez, 2022). Antimicrobial drug resistance (AMR) is accountable for 3.4 percent of new TB infections worldwide and up to 50% of earlier treated patients in certain parts of the globe (Goff et al., 2020; Harding, 2020). The Mycobacteriaceae family includes the genus Mycobacterium, composed of more than 200 species with diverse host reservoirs, varying degrees of pathogenicity in animals and humans, epidemiology, and management despite sharing some basic features (Kanabalan et al., 2021). Mycobacterium can be classified into two distinct categories based on their growth rates: slow-growing Mycobacteria and fast-growing Mycobacteria. For instance, fast-growing bacteria, such as *Mycobacterium smegmatis*, are recognized as opportunistic or non-pathogenic bacteria, while slow-growing Mycobacteria, including *M. bovis*, *M. tuberculosis* (*Mtb*), and *M. leprae*, cause bovine tuberculosis (BTB), human TB, and leprosy, respectively (Forrellad et al., 2013). *Mycobacterium tuberculosis* complex (MTBC) group causes various diseases such as TB and leprosy, skin infections, contributing to increased mortality and morbidity globally. The Mycobacterial species has long been recognized as holding a substantial influence on various sectors, including trade and health, notably TB in humans and other animals. These species can be distinguished from one another through insertional or deletional mutations; however, they are considered to share a common ancestor under evolutionary biology (Pereira et al., 2020).

**Figure 1:**
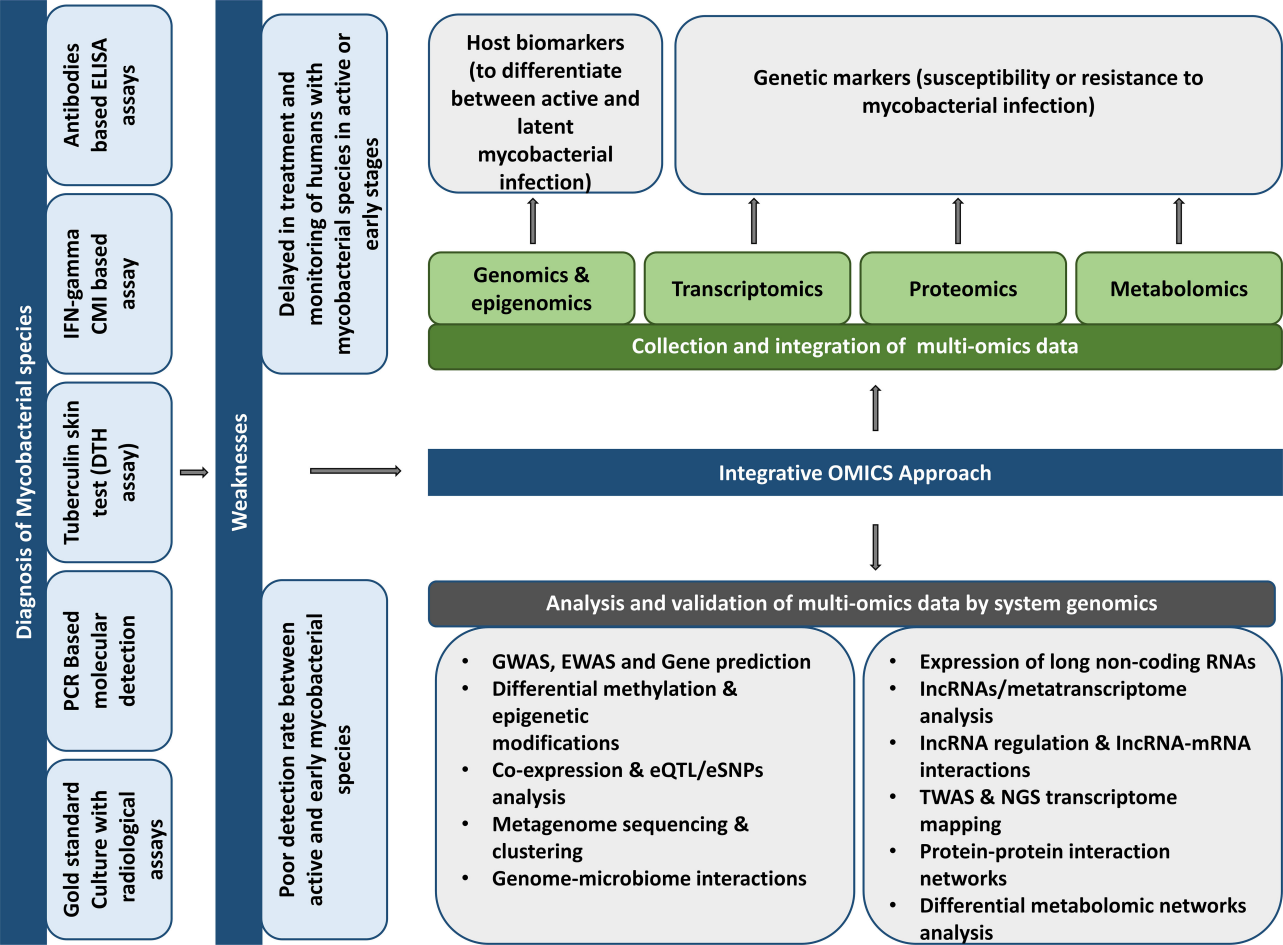
Potentials and suggestions for using different “omics” methods to discover prospective host biomarkers to develop mycobacterial illness diagnostics.

The discovery of new biomarkers and drugs to treat mycobacterial diseases such as TB and leprosy is very challenging. Multiple factors, including bacterial phenotypes, the lipid-rich *Mtb* cell wall as a defense barrier to drug uptake, a slow-growing pathogenic bacterium, drug resistance, drug penetration into bacterial sites, heterogeneity of the clinical disease, the insufficient number of approved drug targets, the demand for the combination of drug therapy, and the length and expense of clinical trials contribute to the difficulty of this task (Goff et al., 2020). Multi-omics techniques are critical in the early disease investigation for novel biomarkers and anti-tuberculosis medicines. Omics methods measure and analyze a class of biological components including DNA, RNA, protein, and metabolites to discover novel targets in druggable pathways for target-based research and characterize the mechanism of action of lead compounds obtained from high-throughput screens. The benefit of multi-omics methods is that they are unsupervised and impartial, making them valuable tools for confirming pharmacological action, elucidating new insights into the compound’s function, and identifying novel biomarkers and pathways for future study (**Figure 1**) (Hasin et al., 2017; Goff et al., 2020).

In this complete review article, the use of multi-omics techniques, such as genomics (DNA), transcriptomics (mRNA), proteomics (proteins), metabolomics (metabolites), and lipidomics (lipids), will be discussed as a framework for developing biomarkers for mycobacterium disease. Multi-omics may provide a comprehensive view of dynamic protein-protein interactions and host-bacterial defense regulation. These insights could significantly contribute to the identification of host biomarkers. Multi-omics approaches have the potential to identify precise host response biomarkers efficiently. These biomarkers may be well-defined biosignatures unique to *Mtb and M. leprae* infections.

## Mycobacteria, Hosts and Diseases

The mycobacterium genus possesses a slightly curved or straight rod morphology (0.2-0.7 x 1.0-10 µm); however, the mycobacterial shape within alveolar macrophages changed from shorter ovals measuring about 0.5 to 1 µm to traditional rods of around 2-4 µm in length and long filamentous forms measuring over 6-7 µm in length (Ufimtseva et al., 2019). Mycobacterium is non-spore producing, aerobic to microaerophilic, and show the actinomycetes group characteristics such as complex cell wall envelope and differential staining procedure known as Zhiel-Neelsen acid-fast stain (Forrellad et al., 2013). The genus includes obligate parasites, opportunistic pathogens, and saprophytes. Based on mycelia type colonies, Lehman and Newman coined the term ‘Mycobacterium’ that exhibits different nutritional requirements and ranges of virulence. Aerobic and chemo-organotrophic mycobacteria show prolonged growth and form visible colonies at optimal temperature in 2-60 days, which may be pink, orange, or yellow depending on light exposure and pigment. Some species show fastidious, require special supplements and take more culture-time to grow than others, e.g., *M. avium* subspecies *paratuberculosis* (Singh et al., 2013) and non-cultivable (*M. leprae* and *M. lepromatosis*); however, it closely resembles *Mtb* (Singh et al., 2015b). Mycobacteria have high, 62-70%, G+C DNA content and a lipid-rich cell wall composed of mycolic acid (C60-C90) and dehydrated menagenunones. Around 40 species of mycobacteria are associated with human diseases that usually produce slow disease, especially slow-developing destructive granuloma that many undergo necrosis with cavitation or ulceration. Genus Mycobacterium is generally non-motile and produces no endospores. Although *M. marinum* is motile inside macrophages, and *M. marinum* and *M. bovis* produce spores (Brennan and Nikaido, 1995; Parish and Stoker, 1998).

In the present situation, *Mtb*, *M. bovis*, *M. leprae*, and *M. lepromatosis* are the most common and causative agents of human TB, bovine TB, leprosy (Hansen’s disease), and diffuse lepromatous leprosy, respectively (Singh et al., 2015b). TB infection generates various host immunological responses that are potentially reliant on host genetic factors and are the primary source of disease susceptibility (Sinha et al., 2014). Other non-tuberculosis mycobacteria, *M. leprae*, a remarkably non-toxic bacteria, cause leprosy, a severely debilitating and stigmatizing nerve disease such as demyelination of the nerve. Anti-myelin basic protein (MBP) autoantibodies generated against peripheral nerves are responsible for pathological degradation of the myelin sheath. The host’s immunological response to *M. leprae* causes most tissue and neurological damage in leprosy patients result in *M. leprae*-specific hypersensitivity responses, such as type-1 (reversal) reaction (T1R) and erythema nodosum leprosum (ENL) (Singh et al., 2015a; de Macedo et al., 2018). Furthermore, *M. bovis*, a zoonotic bacterium that causes incurable tuberculosis in animals and humans, represents severe global health threats as multi-drug and pan-drug resistance strains grow more frequently in the host (McNees et al., 2015; Olea-Popelka et al., 2017)

Hosts and pathogens have evolved new strategies for their growth and survival. Fundamentally, bacterial pathogens search for food or energy for their growth, survival, and reproduction. *Mtb* uses an altered metabolic pathway as a weapon to survive for a prolonged period within the host. In contrast, the host actively depletes nutrients from the intracellular space and pathogen-containing vacuole. This process is called nutritional immunity (Berney and Berney-Meyer, 2017).

**Figure 2:**
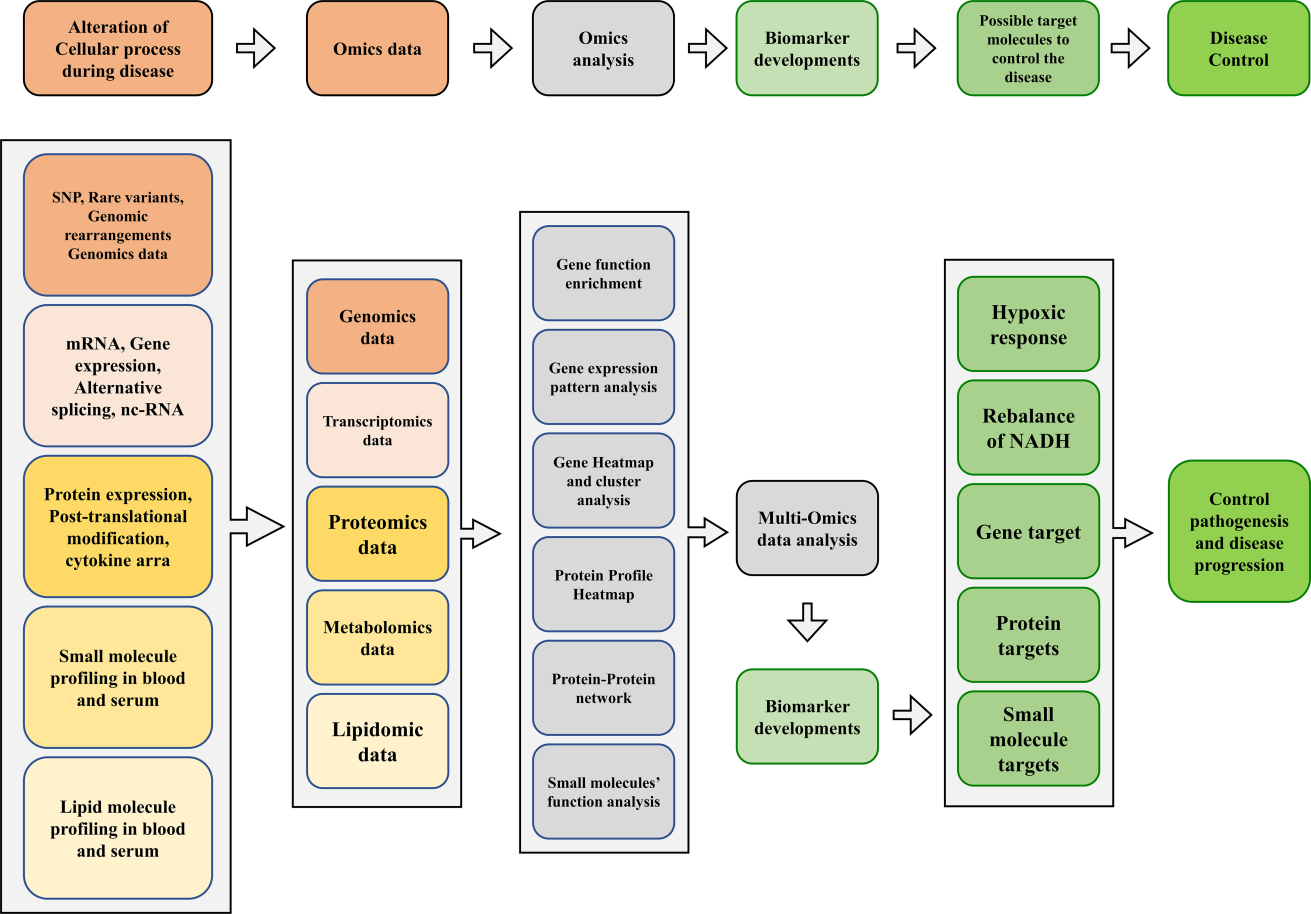
A proposed multi-omics framework and novel biomarker discovery for disease/progression control.

Consequently, the host undergoes several changes during infection at the genomic, transcriptomic, and proteomic levels. In addition, the host has developed many metabolic strategies to limit nutrients during infection and bacterial growth. In addition, amino acid and cofactors metabolism are the primary requirements for the virulence of *Mtb* and other pathogenic mycobacteria. Such pathways could potentially be suitable targets for antimicrobial therapies (**Figure 2**).

## Multi-Omics Approaches in the Perspective of Host-Pathogen Interaction

Current diagnostics for understanding complicated host-*Mtb* interactions are insufficient, while omics-based techniques are more accurate in predicting a more comprehensive picture of mycobacterial disease outcomes. Research advancing related to genomics, transcriptomics, proteomics, metabolomics, lipidomics, glycomics, and glycoproteomics provide additional pathways for investigating the fundamental biology of *Mtb* infection. Earlier studies reported that a systems-level approach based on a two-way proteome microarray strategy could quickly provide useful proteome-wide information and identify novel *Mtb*-Human interactions using 1H nuclear magnetic resonance (NMR) spectroscopy, proteome microarray and mass spectrometry (MS) (Zhou et al., 2013; Lau et al., 2015). Specific *Mtb*-derived glycolipids, mycolic acids and resolvins like metabolites are attractive diagnostic markers for MTBC infection and may modulate host-pathogen interactions, pathogenesis, and resolution (Frediani et al., 2014). Moreover, microRNAs (miRNAs) are a class of highly conserved, single-stranded RNA signatures that correspond to novel biomarkers for TB disease and regulate the expression of target mRNAs (Miotto et al., 2013). Researchers investigated protein N-glycosylation regulation in macrophages and their secreted microparticles useful in intercellular communication during MTBC infection. The modulation of glycosylation enzymes, their receptors and the N-glycome in *in-vitro* differentiated macrophages during *Mtb* infection can be studied using LC-MS/MS-based proteomics and glycomics techniques (Hare et al., 2017).

## Multi-Omics Approaches to Study Mycobacterial Diseases

Previously, the culture technique was the gold standard for diagnosing TB patients, although it is time-consuming and labor-intensive and other techniques for diagnosing TB possess low sensitivity and specificity (Pandey et al., 2016). Furthermore, the aforementioned mycobacterial species discovered several strategies to build a viable human habitat, including modulating host immunological response. Current diagnostic techniques for detecting these invaders are insufficient and have numerous flaws (Thomas et al., 2021). However, all of the knowledge on the pathways and components responsible for these microbes’ detrimental effects has come from pure culture strain studies, which provides little insight into positive results (Lagier et al., 2015). There is an urgent need to create innovative multi-omics technologies that can represent the abilities and activities that those particular microbes can do. “Multi-omics” strategies such as genomic, transcriptomic, proteomic, and metabolic could be a broad strategy to investigate the pathogenesis of mycobacteria in humans and animals (**Table 1**). Multi-omics approaches could offer significant discovery at the early stage of disease in the host.

**Table 1:** Advantages and disadvantages of the analytical techniques employed in omics research.

Omics approaches	Strengths	Weaknesses	Recent improvement	References
**Genomics and Epigenomics**	High throughput sequencing technique Provide static link to the organism Reference genome databases are obtained for reconstruction Gene of interest can be represented in the form of static image	Activity of sequenced genetic element cannot be determined Reconstruction of genome found difficult using bioinformatic software Short read sequencing produce “hard to sequence” intervals	Third generation sequencing Facilitate genome sequencing along with epigenetic determination Higher throughput for shotgun meta-genomics	(Bovee et al., 2008; Sims et al., 2014; van Dijk et al., 2018)
**Transcriptomics**	Provide effective combination with single cell techniques Ample information about data is produced Broad information about environment specific requirement of microbe	Handling errors are accepted during RNA isolation and sequencing RNA restricted technique which provides only snapshot for requirement of the organism RNA’s existence does not strictly forecast the translation into proteins	Higher throughput Next Gen Sequencers (NovaSeq 6000) Provide meta-transcriptomics approach for large systems More reliable software for variant determination and integration	(Lohse et al., 2012; Vogel and Marcotte, 2012; Dagogo-Jack and Shaw, 2018)
**Proteomics**	known protein database predict its relatable functioning provide direct link between organism phenotypic characteristics and proteomic profile provide more stable snaps of organism requirement than comparatively other omics approaches	throughput capabilities do not keep pace with other omics technologies protein research with MS machinery is quite expensive vast array protein analysis get exemption as splitting large protein molecule into small one to facilitate MS analysis	Orbitrap Mass Spec recently added to provide ionization of immensely complex proteins liquid chromatography in combination with multiple MS’s facilitate accurate description of specific groups of proteins PECAN evolved as powerful analytical tool to facilitate accurate predictions specifically from untargeted proteomics	(Pascal et al., 2008; Michalski et al., 2012; Consortium, 2015; Ting et al., 2017; Monaci et al., 2018)
**Metabolomics**	Interlink phenotypic characters with metabolomic profile of organism Provide snapshot for already studied metabolite simultaneously Provide variety of applications across many fields	Sampling artifacts are accepted due to transient nature of metabolites Machine processing is expensive (LC/GC and MS)	High resolution of specific groups is attained with LC or MS High Temperature-Ultra High-Performance LC (LC-MS/MS) are facilitating detection of previously complex metabolites single cell sorting advances are presenting hope for robust and more accurate single-cell metabolomics in the nearest future	(Wang et al., 2014; Chetwynd et al., 2015; Rebollar et al., 2016; Ferrocino and Cocolin, 2017; Zhang and Vertes, 2018)

### Genomics Approaches for Pathogenic *Mycobacterium genus*

The biological system relies on a central dogma (DNA-RNA-Protein) that determines the characteristics and phenotype of any cell species (Franklin and Vondriska, 2011). The first efforts at molecular typing of *Mtb* focused on finding *Mtb*-specific nucleic acids using amplification methods and discovering gene mutations through sequencing. Early investigations employed probe-based typing approaches such as IS6110-RFLP (genetic fingerprint) and PFGE typing to distinguish *Mtb* and *M. bovis* (BCG strain) strains. Second-generation genomic techniques for *Mtb* molecular typing include mixed-linker PCR (ML-PCR), fast ligation-mediated PCR (FliP), and ligation-mediated PCR (LM-PCR) (Jagielski et al., 2014). Furthermore, spoligotyping based on direct repeats loci and MIRU-VNTRs based on mycobacterial interspersed repetitive units have been utilized for phylogeography of the *Mtb* complex (Bouklata et al., 2015). Other technologies, such as GeneXpert (cartridge-based nucleic acid amplification assay), may identify *Mtb* genetic alterations linked with rifampicin resistance (Bunsow et al., 2014). Third-generation sequencing technologies evolve a series of steps incorporated with DNA extraction, its amplification, sequencing, structural and functional annotation of the genome, which is now successfully used to identify prospective products derived from the genetic code and their viable pathways (Heather and Chain, 2016). Comparative genomic research between the generic pathways of available Mycobacterium species provided information about the unique functional capabilities of the particular species strain with special reference to the substructure of potential drug targets (Ilina et al., 2013). High-throughput gene expression technologies have revolutionized medical research and are mainly driven by technological advances that have positively impacted the cost-efficient, high-throughput analysis of biological molecules (Goff et al., 2020). Moreover, Array-based comparative genomics demonstrated the potential approach for retracing microbial evolution, molecular epidemiology, and pathogenesis. A study based on high-density oligonucleotide microarray demonstrated that epidemiologically and clinically characterized *Mtb* can be utilized for genetic variability among the natural population and detect trim-level genomic deletions (Kato-Maeda et al., 2001).

#### Comparative Genomics of Vaccines by Whole-Genome DNA Microarray

The Behr group investigated the genomic compositions of *M. bovis*, *Mtb*, and several BCG (Bacille Calmette-Guerin) daughter strains utilizing comparative hybridization on a DNA microarray. With the aid of sequencing across the missing section of the reference strain’s genome, deleted regions from BCG vaccinations were established relative to *Mtb* H37Rv reference strains. In this study, eleven H37Rv regions were absent from one or more virulent *M. bovis* strains; however, five more *M. bovis* regions were missing from some BCG strains, indicating that BCG strains have been evolving since their inception. This thorough understanding of genetic diversity across closely related mycobacterial species gives reasonable ways for developing the groundwork for improved diagnostic approaches and vaccines (**Table 2**) (Behr et al., 1999). Expression quantitative trait loci (eQTL) were verified as significant in explaining genome-wide association studies (GWAS) to identify single nucleotide polymorphisms (SNPs), genes at GWAS loci, and investigate drug resistance-related mutations (Nica and Dermitzakis, 2013). An Illumina whole-genome sequencing study revealed the minimum inhibitory concentration (MIC) for 12 anti-TB drugs when tested together on 1452 clinical *Mtb* isolates and the genome-wide associations between mutations in the non-coding region of *Mtb* genes and resistance. This study was verified using a data collection of 792 patient isolates, and the findings revealed correlations at 13 non-canonical loci with two non-coding areas (Farhat et al., 2019).

**Table 2:** Comparative genome characterization and synteny of pathogenic mycobacterial species and their strains.

Characteristics	*Mycobacterium tuberculosis* H37Rv	*M. avium* 104	*M. avium* subsp. *paratuberculosis* K10	*M. avium* subsp. *paratuberculosis* S5	*M. avium* subsp. *paratuberculosis* MAP4	*Mycobacterium lepare*	*Mycobacterium lepromatosis*
**Size (bp)**	4,411,532	5,475,491	4,829,781	4,798,157	4,829,424	3,268,210	3,215,823
**GC content (%)**	65.61	68.99	69.30	69.25	69.30	57.79	57.89
**Protein coding genes**	4,018	5,120	4,350	4,288	4,326	1,614	1,477
**Functional assigned**	3,507	3,547	1,231	1,657	3,193	308	143
**Hypothetical**	511	1,573	3,119	2,631	1,133	1,306	1,334
**tRNAs**	45	46	45	46	46	45	45
**rRNAs**	3	3	3	2	3	3	3

Most GWAS are conducted on African populations; however, there is potential to discover new targets in genetically different populations that might be beneficial for conducting multi-stage GWAS in an Indonesian cohort (Peprah et al., 2015). Initially, DNA microarrays were helpful in mapping the fluctuating abundance of transposon (Tn) mutants and transposon site hybridization (TraSH), which has recently been combined with whole-genome sequencing (Tn-seq) to achieve volumetric genomic resolution. Tn mutant *in-vitro* screening is a valuable technique for identifying genetic pathways involved in the development of *Mtb* and *M. bovis* (DeJesus et al., 2017). Jian’s group started a genome-wide transcriptomics approach that provides the first insights into *Mtb* reaction to the coumarin derivative Osthole (7-methoxy-8-isopentenoxycoumarin) from medicinal plants, as well as the anti-mycobacterial effect of Osthole (Wei et al., 2013). Previous research built phylogenetic trees based on core proteins for 150 species, including the genus Mycobacterium, Actinobacteria, and others, and introduced new omics techniques known as comparative genomic analysis with phylogenomics analyses on mycobacterial genomes to describe their interrelationships (Gupta et al., 2018). Another research demonstrates the first whole-genome analysis of a Mycobacterium sp. UM CSW was isolated from a bronchiectasis patient using comparative genomics, molecular phylogenetic, ANI, and AAI studies (Choo et al., 2016).

#### Gene Chips and CRISPR Interference (CRISPRi) Techniques for the Diagnosis of Mycobacterial Diseases

Investigations revealed that instead of traditional methods such as tissue inspection under a microscope, genetic profiling could diagnose and classify leprosy infections correctly. Gene expression profiling showed significant differences in gene expression and classified the disease’s clinical type (King and Sinha, 2001). Further investigation suggests that DNA microarrays and gene chips are viable approaches for monitoring the activity patterns of different immune-system genes throughout various stages of the illness. As a result, gene expression profiles define the clinical form of the illness and provide a better knowledge of how immune responses to pathogens are regulated. In addition, more active genes stimulate killer T cells, known to fight invading infections (Bleharski et al., 2003; Zheng et al., 2020).

Moreover, single guide RNA (sgRNAs) libraries were created using Clustered Regularly Interspaced Short Palindromic Repeats interference (CRISPRi), a high throughput screening method. CRISPRi (dCas9-sgRNA complex) disrupts RNA polymerase promoter access, destabilizes the DNA duplex, and inhibits gene transcription. Thus, gene silencing of *Mtb* strains by CRISPRi can identify and validate therapeutic targets (Goff et al., 2020). Many genes, which are CRISPRi targets and allow for high-throughput screening techniques, were used to create libraries comprising more than 90,000 sgRNAs and create pools of various *Mtb* strains. S. thermophilus CRISPRiCas9 (Sth1Cas9) systems have recently been used in *Mtb* and other NTMs to enable gene editing and effective CRISPR interference-mediated transcriptional regulation (Meijers et al., 2020). Hence, the advancement of next-generation sequencing technologies, including Illumina sequencing (short sequence reads), PacBio (long-read sequencing), and Oxford Nanopore (structural variation and sequencing repetitive regions), as well as decreasing technology costs over time, have allowed for the incorporation of whole-genome sequencing into the drug discovery pipeline for mycobacterial diseases (Niedringhaus et al., 2011).

### Transcriptomics Approaches for Pathogenic Mycobacterial Species

The transcriptomics approach involves collecting an RNA expression profile, which is highly dynamic compared to constant genome profiling and employs gene expression, microarray, RNA sequencing (RNA-seq), digital profiling, and serial analysis of gene expression in microbes (Lowe et al., 2017). Transcriptomics research provides information for mycobacterial responses and understanding the mechanism of pathogenicity and therapeutic action. It also finds novel drug targets and assigns gene functions. It is also an essential technique for detecting pathogenic mycobacterium species (Boshoff et al., 2004). Furthermore, methods such as RNA-seq, comparative microarray, next-generation sequencing (NGS), and cDNA suppression subtracted hybridization (SSH) are appropriate for studying the whole transcriptome of pathogenic mycobacterial species. Recently, RNA-seq gene expression studies have shown the ability to capture pictures of the differential expression of transcripts seen in mycobacterium species (Chung et al., 2021). Previous research revealed a new transcriptomic method that used overlapping of chromatin immunoprecipitation sequencing (ChIP-seq) RNAP (RNA Polymerase), and NusA RNA-Seq data to detect sRNA expression in *Mtb*. In addition, many sRNAs, including ncRv11806 and DrrS, were expressed in the stationary phase, suggesting their relevance to mycobacterial latency and long-term pathogenicity (Ami et al., 2020). Furthermore, an in-silico study using the INDIGO-*MTB* computational model revealed that *Mtb* transcriptomic signatures following drug exposure were examined in over a million potential combinations of 164 drugs that predict antagonistic and synergistic efficacy of 35 existing potential anti-TB drugs (Ma et al., 2019). Moreover, a single-cell transcriptomics study of *Mtb* revealed phenotypic diversity, including within and across infected individuals. For instance, a transcriptome-wide study of *Mtb*-specific cells in latently infected people revealed variations in the transcriptional phenotypes of *Mtb*-responsive CD4^+^ T-cells within and across people. This research proposed possible vaccine targets and disease-fighting mechanisms (Burel et al., 2018; Kirschman et al., 2020). Furthermore, single-cell RNA sequencing (scRNA-seq) in TB showed depletion of the natural killer (NK) cell subset in TB patients (Cai et al., 2020).

In addition, *in-vivo* methods such as high-throughput RNA sequencing coupled with hypothesis-generating methodologies and High-Resolution Transcriptomic Analysis by Whole-Transcriptome Sequencing may help researchers better understand mycobacterial species’ pathogenic processes (Benjak et al., 2015). Dual RNA-seq research investigated the etiology of leprosy illness and discovered a link between bacterial load, transcriptional status, and the host immune response system inside leprosy skin lesions (Montoya et al., 2019). Blood transcriptomic biomarkers research recently evaluated four blood RNA signatures, including MT-ND2, REX1BD, TPGS1 and UBC collectively known as RISK4LEP, which can predict leprosy years before clinical onset and could allow for early diagnosis, better treatment, and the prevention of bacterial transmission (Tió-Coma et al., 2021). Furthermore, single-cell and spatial transcriptomics in leprosy granulomas characterize the antimicrobial response network. The transcriptomics research found that genes encoding proteins needed for antimicrobial responses are differently expressed in reversal reactions (RR) vs lepromatous leprosy (L-lep) lesions and are controlled by IFN-γ and IL-1β. A map of leprosy biopsy specimens was created employing single-cell and spatial sequencing of primary cell types and antimicrobial gene expression in RR and T-lep lesions. These study methods revealed the ordered architecture of granulomas, revealing the compositional and functional layers through which macrophages, keratinocytes, fibroblasts, and T cells contribute to the antimicrobial response (Ma et al., 2020).

#### Role of RNA Sequencing in the Diagnosis of Mycobacterial Diseases

The present state of mycobacterial diseases has compelled global researchers to focus on infection prevention and control, although appropriate techniques, methodologies and the development of more effective vaccines are challenging. The majority of mycobacterial illness diagnoses are based on immunosorbent tests (ELISA) technology. Some subspecies are closely related to one another and interact with indirect diagnostic techniques (antibody ELISA) (Singh et al., 2016). Transcriptomics-based genetic profiling sheds light on the host’s response to infection, cellular function and regulatory mechanisms that can aid in identifying novel biomarkers along with the differentiation of disease-resistant and vulnerable animals (Burel et al., 2019).

Transcriptomics profiling creates a transcriptome by examining a species’ whole genome. The genome generates both coding mRNA (messenger RNA) and non-coding RNAs. These particular RNA segments perform a range of tasks, including amino acid delivery to the ribosome (transfer RNA), gene regulation (small RNA, long non-coding RNA and micro-RNA) and enzyme-like activity (ribozymes) (Statello et al., 2021). The mapping of these RNA strands provides valuable spatiotemporal data on physiological cell state, RNA dynamics and their activities. One of these large-scale methods is microarray: available DNA probes are placed on a chip containing all open reading frames (ORFs), which can measure gene expression levels (Sun and Chen, 2020). RNA sequencing is a method for generating an extensive library of RNA derived from cDNA. RNA profiling of both the host and the pathogen provides a foundation for understanding host-pathogen interactions (Westermann et al., 2017). Deep sequencing has allowed accurate mapping of transcription start, termination sites, amplification of RNA transcription and regulatory mechanisms such as promoter sites (Kukurba and Montgomery, 2015). Transcriptomics can identify possible infectious pathogen antigens such as circulating and secreted host RNA (miRNA, lncRNA),blood cell produced RNA and bacterial secreted RNA (van den Esker and Koets, 2019).

*In-vivo* analysis of mycobacterial species, molecular dynamics revealed that dual RNA sequencing on *Mtb* could ontogenetically distinguish infected macrophage lineages from other human body cells. Mycobacterium-infected mouse lung macrophages revealed a greater level of 180 genes than alveolar macrophages. Thus, *in-vivo* dual RNA-seq revealed that the microbe’s transcriptional response varied across alveolar macrophages (Peddireddy et al., 2017). Comparing the genome, transcriptome and methylome of three main *Mtb* lineages showed that methylation influences genetic mutation, variance in mycobacterium virulence and pathogenicity. Thus, identifying genes associated with drug resistance, efflux pumps (Rv2994 or iniA and iniB), virulence and pathogenicity (vapBC family) may reveal clade-specific variation in ancient and contemporary strains (Pisu et al., 2020).

#### piRNAomics

PIWI-interacting RNAs (piRNAs) are single-stranded (23–36 nucleotide) RNAs that form the biggest category of short non-coding RNAs which are different from siRNAs and microRNAs (miRNAs). These piRNAs have an essential role in post-transcriptional modification, such as mRNA silencing, transposon silencing, epigenetic control and germline development (Wang and Lin, 2021). All piRNAs, except piR-hsa-27283, were shown to be downregulated in leprosy skin lesions. Human piRNAs, like miRNAs, undergo post-transcriptional alteration, including mRNA silencing which are associated with various processes, including apoptosis, epithelial-mesenchymal transition (EMT), *M. leprae* identification, engulfment, loss of neuropathic sensation pain and Schwann cell (SC) demyelination. The piRNA sequences study revealed the critical function of piRNAs in disease processes and offered a novel therapeutic target for precisely controlling nerve damage. Upregulated piRNA (PIR-has-27283) may also be used as a disease biomarker (Pinto et al., 2020). Thus, a biomarker, any structure, activity or substance that may predict an event or illness must be measured and transcriptomic analysis may help identify novel indicators for early infection.

### Proteomics Studies for Mycobacterial Species and Host Biomarkers’ Discovery

The study of proteins expressed in cells, tissues, or organisms is termed proteomics. Proteomics involves three crucial steps: isolation, digestion into peptides, and identification. Various techniques can be employed for these steps, including two-dimensional gel electrophoresis (2DGE) and various chromatography-based procedures. MALDI-TOF or ESI analyzes the peptides resulting from enzymatic digestion. In addition, iTRAQ, a shotgun technique, offers improved reproducibility and sensitivity. Proteins that accumulate or drop in quantity across different proteomes could be potential biomarkers (Gautam et al., 2021). The development of proteomics has gained widespread attention since its inception and now stands at a transitional stage from benchwork to clinical applications. Proteomics profiling identifies target proteins that serve as host biomarkers in disease diagnosis, treatment and prevention (Kanabalan et al., 2021). The two strategies are principally exercised to measure *Mtb* proteins. The first is antibody-based methods, including western blotting and ELISA, and the second is proteome discovery by mass spectrometry (MS). Discovery-driven MS, also called shotgun MS, is the most extensively accepted method for identifying and quantitative measurements of proteins and maximizing proteome coverage. However, the selected reaction monitoring (SRM) approach is the gold standard targeting method, also known as the multiple reaction monitoring (MRM) method. SRM comprises an extensive dynamic range of proteins with accurate reproducibility and has quantitated 97% of annotated *Mtb* proteins (Schubert et al., 2013).

A highly multiplexed proteomic approach (such as SOMAscan, SomaLogic, Inc, Boulder, CO) revealed enrichment for proteins involved in a variety of processes such as inflammation pathways, antimicrobial defense, tissue healing and remodeling, acute phase response, coagulation cascade, apoptosis and immunity. This proteomic approach is utilized to identify appropriate host biomarkers in mycobacterial disease (De Groote et al., 2013). Another research found that serum adenosine deaminase enzymatically converts adenosine to inosine as a possible serum proteomic biomarker for TB that may be utilized to quickly and efficiently diagnose TB (Pandey et al., 2016). Proteome microarray research allowed screening of blood serum from individuals with active TB and latent TB infection and found that RV2031c, RV2421c,and RV1408 were possible serum biomarkers for distinguishing active TB from latent TB infection (Cao et al., 2018). Another serum biomarkers study found that proteins including S100 calcium-binding protein (S100-A9), superoxide dismutase (SOD), a-1-acid glycoprotein 2 (ORM2) and IL-36a were significantly enhanced in patients with acute pulmonary TB. These identified proteins are linked to the transmission of TB and may be utilized to distinguish between tuberculosis phases (Liu et al., 2018).

Moreover, the Label-free quantitative proteomics approach found that patients with pulmonary TB had higher levels of plasma proteins, including alpha-1-antichymotrypsin (ACT), alpha-1-acid glycoprotein 1 (AGP1) and E-cadherin (CDH1) compared to individuals with latent TB infection and healthy controls. These plasma protein indicators can differentiate between pulmonary TB and latent TB infection (Sun et al., 2018). Further research found two predictive proteome biomarkers such as TB Risk Model 5 (TRM5) signature and 3-protein pair-ratio (3PR) signature, which may be utilized to predict the progression to incident TB within a year of diagnosis (Penn-Nicholson et al., 2019). Expression levels and functions of *Mtb* proteins are associated with mycobacterium biology, its infection and host-*Mtb* interactions. Researchers have determined that Tropomyosin-specific peptides produced by *M. leprae* in leprosy patients result in muscle weakness due to the presence of anti-myosin antibodies generated by the host against the peptide. This finding was made using 2-D gel electrophoresis, western blots, and MALDI-TOF/TOF antibody-reactive spots. Therefore, auto-reactions play a role in muscle damage, resulting in losing muscular functioning in leprosy patients (Singh et al., 2018). The quantification of the *Mtb* proteins required in infection and host-*Mtb* interactions is critically important to advance the knowledge of *Mtb* biology during infection, survival and persistence (Schubert et al., 2013). Proteome chips technology can enhance proteomics research in prokaryotes by revealing crucial interactions between proteins and nucleic acids that are tedious and hard to determine using conventional approaches (Chen et al., 2008).

Recent research explored anti-*Mtb* drugs, including bedaquiline (TMC207), gatifloxacin and metronidazole which can eliminate antibiotic-resistant strains and contribute to advancement over existing treatments (Danelishvili et al., 2017). However, the most significant hurdles of anti-TB therapy are that bactericidal compounds concentrations increase the pathogen’s drug susceptibility, resistance power and shifts into different metabolic states to survive. Early proteomic study data revealed that *Mtb* upregulates the synthesis of numerous protein enzymes during exposure to bactericidal compounds or drugs, such as isoniazid (INH), rifampicin (RIF), ethambutol (EMB) and pyrazinamide (PZA) that are utilized “escape” pathways to enhance bacterial survival (Briffotaux et al., 2019). Similarly, *Mtb* increases the expression of the LpqY-SugA-SugB-SugC ATP-binding cassette transporter during drug treatment, which is an essential virulence factor. Trehalose carbohydrate ABC transporters are particular for uptake of the disaccharide trehalose sugar (not present in mammals), which is essential for establishing infection in the host and *Mtb* pathogenesis. Trehalose carbohydrate ABC transporters associated with mycolic acid processing and Trehalose recycling. ABC transporter and trehalose recycling equipped the *Mtb* to bypass glucose phosphorylation and use trehalose as a primary carbon and energy source under nutrient-restricted environments (Kalscheuer et al., 2010; Grzegorzewicz et al., 2012). Thus, the proteomics approach could translate these biomarkers from laboratory to clinical application, and the proteomics approach is expected to show promise as a practical approach in TB diagnosis and exploring treatment biomarkers.

### Metabolomics Research for Mycobacterial Species and Host Biomarkers’ Discovery

The metabolomics approach complements other “omic” sciences such as genomics, transcriptomics and proteomics. The metabolomics approach has fewer restrictions due to technical and biological advantages which may offer a complete understanding of cell activity compared to other omic approaches (Mirsaeidi et al., 2015). Metabolites are low-molecular-weight compounds produced by metabolic processes in living things called metabolism that change over time. Metabolomics involves categorizing and measuring these metabolites which may be used to track disease progression and adaptive processes. The metabolomics approach studies biological fluids such as blood, urine, sputum, CSF and bacterial sources such as culture medium (Mirsaeidi et al., 2015). Previously *Mtb* and TB pathogenesis were described by three terms, latency, persistence, and dormancy. Previous observations have confirmed that “*in-vivo* grown” *Mtb* was metabolically different from bacteria raised *in vitro*. The fundamental requirement of pathogenicity of *Mtb* is metabolic adjustments (Gomez and McKinney, 2004). Mycobacterium such as *Mtb* physiology and pathogenesis depends on metabolic pathways essential for their survival and infection in the host. Recent approaches, particularly metabolomics, are a systems biology tool explaining *Mtb*’s biochemical environment that provides a detailed insight into infection in experimental models. Thus, the metabolic approach clarifies mechanisms of action of new and existing anti-tuberculosis drugs and opens new doors for developing advanced drugs/interferences to counter TB (Warner, 2014). Metabolic adaptations are crucial for *Mtb* pathogenesis. *Mtb* survives in the microbicidal stressed environment, including acidic/low pH, hypoxic and redox stresses, reactive oxygen, nitrogen intermediates and shortage of vital micronutrients within the host alveolar macrophages of the lungs. In response to hypoxia, *Mtb* shows widespread metabolic changes in various metabolic pathways, including cholesterol catabolism, methyl-branched lipids, the metabolism of triacylglycerides (TAG) and extensive alterations in both intracellular and extracellular amino acid levels. Under restricted nutrient availability, *Mtb* shifts to lipids and host-derived cholesterol as primary nutrient sources (Garay et al., 2015). Numerous investigations have revealed metabolic alterations in macrophages during *Mtb* infection. For instance, gas chromatography-mass spectrometry analysis explained an abundance of amino acids such as glycine, aspartate, proline, isoleucine, alanine, ornithine, threonine, cysteine and lysine were reduced. In contrast, amino acids including glutamate, serine and valine were raised in *Mtb*-infected macrophages (Cheng et al., 2013). Furthermore, lepromatous patients with ENL (type 2 responses) exhibited higher nitric oxide metabolites in their urine. When phagocytic cells such as macrophages and neutrophils come into contact with a pathogen, they generate reactive nitrogen intermediates (RNI) such as nitric oxide (NO) through activation of nitric oxide synthase (iNOS) to control the immune response and decrease inflammation. Moreover, serum nitrite levels were significantly higher in individuals with tuberculoid leprosy than those with lepromatous leprosy (Mohanty et al., 2007; Dubey et al., 2020).

Che et al. utilized gas chromatography/time-of-flight mass spectrometry (GC/TOF-MS) to identify blood serum metabolite biomarkers associated with active TB and discovered that 5-oxoproline levels were consistently lower in active TB patients (Che et al., 2013). The technique of liquid chromatography high-resolution mass spectrometry (LC-MS) is used to identify metabolites in plasma samples from people with active TB, their asymptomatic household contacts and identify new pathophysiologic pathways involved in the progression and resolution of TB infection. This research discovered that metabolite clusters such as anti-TB medications, glutamate, choline derivatives, *Mtb*-derived cell wall glycolipids (trehalose-6-mycolate and phosphatidylinositol) and resolvins were mainly raised in TB patients and could be used as biomarkers (Frediani et al., 2014). Moreover, the metabolomics study utilized ultrahigh-performance liquid chromatography-electrospray ionization-quadrupole time of flight mass spectrometry (UHPLC-ESI-QTOFMS) to identify potential biomarkers for diagnosing TB and discovered that four metabolites including ceramide, 12R-hydroxy-5Z, Z,10E,14Z-eicosatetraenoic acid [12(R)-HETE], cholesterol sulfate and 4-formyl-4-methyl-5-cholesta-8-en-3-ol. These new plasma biomarkers, particularly 12(R)-HETE and 4-formyl-4-methyl-5-cholesta-8-en-3-ol may be suitable for fast and non-invasive detection of TB (Lau et al., 2015). Furthermore, the Collins et al. group developed high-resolution metabolomics (HRM) techniques that use liquid chromatography and ultra-high-resolution mass spectrometry (LC-MS) to detect putative biomarkers in plasma metabolites and other biosamples of TB patients. This research discovered that patients with active TB had a significantly higher level of *Mtb*-associated metabolites, including acylphosphatidylinositol mannoside (Ac1PIM1), lysophosphatidylinositol (Lyso-PI) and phosphatidylglycerol (PG) than their household contacts (Collins et al., 2018).

Mass-spectrometry-based metabolomics research showed that drug-susceptible (DS), multi-drug-resistant (MDR) and extensively drug-resistant (XDR) *Mtb* strains possess different metabolic profiles. Ultra-High-Performance Liquid Chromatography and High-Resolution Mass Spectrometry studies showed that amino acids, including isoleucine, betaine, proline, and pantothenic acid, altered significantly between strains with different drug susceptibility profiles (Rêgo et al., 2021). A targeted metabolomics profiling was performed using liquid chromatography-tandem mass spectrometry (LC-MS/MS) followed by multivariate and univariate analysis to search for potential biomarkers in patients with active TB, latent TB infection (LTBI), and healthy controls. According to the findings of this study, active TB patients had higher blood levels of aspartate, glutamate, methionine and sulfoxide while lower serum levels of asparagine, glutamine and methionine were observed compared to LTBI patients or healthy controls. As a result, new serum biomarkers including glutamate, sulfoxide methionine, aspartate, glutamine, methionine and asparagine may help detect adjunctive, quick and non-invasive pulmonary TB (Cho et al., 2020). Genome-scale metabolic models explain the correlation between genes, proteins, and enzymes in the organism and process the information from both computationally predicted biochemical processes and experimentally validated processes within the animal. Flux balance analysis (FBA) predicts a network of metabolic capabilities at a steady-state and predict gene knockouts’ metabolic phenotype, metabolite uptake and secretion rates over time (Orth et al., 2010). An extended FBA methods called E-Flux and E-Flux-MFC methods translate gene expression data. E-Flux method has been used to predict the consequence of drugs on *Mtb* mycolic acid biosynthesis. E-Flux-MFC has been used to precisely predict variations in the generation of both external and internal metabolites by combining gene expression data and prophesying alterations in lipids and metabolites during hypoxia over the time course (Garay et al., 2015). A new Probabilistic Regulation of Metabolism (PROM) method enables automated, straightforward, and quantitative integration of high-throughput data into soft constraints on model reaction rates. E-Flux is used to predict terminal, or sink metabolites, whereas PROM is flexible for studying those same metabolites (Chandrasekaran and Price, 2010).

Consequently, metabolomics has enhanced our understanding of many mycobacterial diseases molecular processes and offered a platform for discovering novel biomarkers. Metabolites from different biological samples may be potentially fast, complementary, and non-invasive biomarkers in diagnosing and monitoring mycobacterial diseases. However, comprehensive metabolite assessment demands highly specialized and skilled scientific approaches and excellent bioinformatics investigations, which remains a significant hurdle in low-resource areas where TB is common.

### Lipidomic Investigations for Pathogenic Mycobacterial Species

Lipids are small molecules like other significant biomolecules, including nucleic acids, polysaccharides and proteins. Lipids are produced from anabolic and catabolic reaction pathways and digested by enzymes affected by the environment of a particular biological system, such as food, temperature and pressure (Wenk, 2010). Lipidomics, a lipid-targeted metabolomics approach focusing on a comprehensive investigation of all lipids, gives insights into the precise functions of lipid species in health and disease conditions and recognizes potential biomarkers for the development of preventive or therapeutic programs for mycobacterial infection (Zhao et al., 2015). Because of the complexity of lipids and a lack of reliable instruments for their study, understanding lipidomics is incomparable to genomics and proteomics research. Novel lipid analysis techniques, such as liquid chromatography and mass spectrometry, have been extensively utilized in lipidomics research (Wenk, 2005). For instance, fatty acids such as Mycolic acids (MAs), α-alkyl, β-hydroxy long-chain fatty acids modulate host innate immune responses and create an effective permeability barrier. MAs are abundant in the cell envelope of MTBC and are the targets of many anti-tuberculosis medicines. According to mass spectrometry studies, there are substantial differences in MA patterns across various MTBC strains and lineages (Portevin et al., 2014).

Moreover, Omega-3 and omega-6 fatty acids generate lipid mediators such as cysteinyl leukotrienes, leukotriene B4, prostaglandin E2 and D2, lipoxin A4 and resolvin D1. These lipid mediators are involved in regulating *M. leprae*-specific inflammatory and immunological responses. The levels of lipid mediators may be determined using liquid chromatography-mass spectrometry-mass spectrometry (LC-MS-MS) or enzyme immunoassay (EIA) kits, which are both highly sensitive but have varying degrees of cross-reactivity (Amaral et al., 2013; de Macedo et al., 2018). Ultra-performance liquid chromatography-mass spectrometry (UPLC-MS) study found altered host lipid metabolism and changed high-density lipoproteins (HDLs) compositional and functional profiles in Multibacillary (MB) leprosy patients. MB pre-MDT patients have shown an altered Apoprotein A-I (ApoA-I), the primary HDL protein compared to a healthy and a post-MDT recovered individual (Lemes et al., 2020).

The single-stage and tandem mass spectrometry approaches showed that amphiphilic lipids, including glycerophospholipids, sterols and sphingolipids, constitute human cell membranes where they are unevenly distributed. In addition, particular lipids are supplied in specific organelles, such as lysobisphosphatidic acid (LBPA)/bis (monoacylglycerol) phosphate (BMP) is primarily enriched in endosomal/lysosomal membranes and cardiolipin (CL) a phospholipid, which is exclusively located in mitochondria (Wenk, 2010). Mycobacterial species are unique and possess a unique capability for synthesizing a wide array of hydrophobic lipids and secondary metabolites. Earlier investigations validated that the *Mtb* genome contributes a powerful lipid biosynthetic capacity to the bacterium, resulting in abundant lipids on the surface, forming a complex cell wall that provides defensive functions to the bacterial cell. Alterations in lipid content enable the bacteria to adjust to diverse stresses and infections (Chow and Cox, 2011). After entry into the host cell, *Mtb* utilizes host lipids as a primary nutrient source. *Mtb* infection regulates TB patients lipid metabolism and favors the degradation of phospholipids and accumulation of cholesterol esters, resulting in cavities with caseous necrosis in the lungs (Han et al., 2021). *Mtb* also utilized lipids in the development of multi-drug resistance (MDR). The importance of *Mtb*’s lipid profile could be understood by the bacterial genome, which has a 30% genome dedicated to encoding the lipid. Electron microscopy research has shown that drug-resistant *Mtb* strains have thicker cell walls than non-resistant *Mtb* strains (Pal et al., 2017).

The gold standard for diagnosing mycobacterial diseases such as leprosy is a skin biopsy, which is invasive and has poor sensitivity. The more sophisticated direct-infusion electrospray ionization high-resolution mass spectrometry (ESI-HRMS) approach was utilized to detect lipid markers in leprosy patients directly from skin impressions (Amaral et al., 2013; Lima et al., 2015). Matrix-assisted laser desorption-ionization imaging mass spectrometry is a powerful technique for localizing and characterizing lipids in biopsy tissues (de Macedo et al., 2015). The high-resolution mass spectrometry (MS) or liquid chromatography-mass spectrometry (LC-MS) approach provides a lipidomic profile of *Mtb* with some limitations, including mass spectra peak overlap, some lipid species are not detected and lack of a lipid database synthesized by mycobacteria. However, the LIPID MAPS consortium of lipidomics researchers has created a robust database including common lipids such as glycerophospholipids and triacylglycerols. Although, these databases do not comprise the unique mycobacterial lipids data, such as sulfolipids and phenolic glycolipids (Chow and Cox, 2011). MycoMass, a potent lipid database for the study of *Mtb*, is established by Layre et al. group, which is like LIPID MAPS. MycoMass database supports detecting the dynamic shifts of mycobacterial species lipids during infection and understands lipids’ role in virulence (Layre et al., 2011). Another ultra-high-performance liquid chromatography-tandem mass spectrometry approach allows for screening of plasma lipids in TB patients that provides different analyses, including principal component analysis, orthogonal partial least squares discriminant analysis and K-means clustering algorithm analysis to identify lipids with differential abundance. This approach demonstrated that TB patients had increased cholesterol levels and decreased plasma phospholipid levels. This study confirms that lipids such as phosphatidylcholine, cholesteryl ester and sphingomyelin are promising biomarkers for the early detection of TB (Han et al., 2021).

In addition to nutrients, *Mtb* needed micronutrients from the host. These micronutrients such as iron are essential for the growth and pathogenesis of bacteria. Conventional TLC and advanced mass spectrometry-based LC–ESI–MS techniques were used to discover changed lipidome patterns. Lipidome analysis reported alterations in lipid profiles significant for pathogenicity and exhibited the necessity of micronutrients such as iron to sustain metabolic, genotoxic and oxidative stresses (Pal et al., 2019). Thus, a comprehensive lipidomic approach could prevent the *Mtb* turn into MDR. The high-throughput mass spectrometry-based lipidomic approach can differentiate the lipidome profile of drug-sensitive (DS) and resistant (DR) strains of *Mtb* and has been declared that lipids including fatty acyls (FA), glycerophospholipids (GPL), and glycerolipids (GL) play significant roles in *Mtb* drug resistance (Pal et al., 2017). High-performance liquid chromatography (HPLC) and thin-layer chromatography (TLC) were early lipidomics methods that were time-consuming and lacked sensitivity. Although these methods may describe global alterations across lipid classes, they cannot detect precise remodeling of specific molecular species within a single lipid class. Several appropriate novel techniques, including gas chromatography–MS (GC-MS), liquid chromatography–MS (LC-MS) and NMR are widely employed to profile lipid repositories (Layre et al., 2014). Undoubtedly, more new features and functionalities are needed to add-in currently utilized in lipid MS analysis. For example, ion mobility mass spectrometry (IM-MS) can identify lipids directly from tissue slices using MALDI. ICP mass spectrometry is a suitable technique for imaging lipids that bind to cations such as Ca^2+^ and Mg^2+^ through their charged head groups (Becker and Jakubowski, 2009). Coherent anti-stokes Raman scattering (CARS) microscopy is a fascinating new method for lipid imaging that has just been created. As a result, CARS is fast, does not require external labeling and may be utilized for real-time imaging (Wenk, 2010). The composition of *Mtb* lipidome is still unknown. Consequently, new technologies could enhance our understanding of the lipidomics approach to understand many mycobacterial diseases and provide a platform for discovering novel lipid biomarkers.

## Conclusion and Future Prospects

This review focused on current mycobacterial omics technologies and their application to determining mycobacterial signatures during the disease’s early stages. Mycobacterial signatures such as proteins, metabolites, and transcript biomarkers are more likely to identify active TB. Current multi-omics techniques suggest that leveraging the host response to detect mycobacterial diseases like TB and leprosy may be feasible despite the ineffectiveness of prior diagnostics. Additionally, this review addressed omics techniques and their applications for resolving the complexity of proteins, metabolites and transcript networks involved in infection and bacterial physiology. When combined with an understanding of bacterial gene regulation, metabolism, adaptability, and pathogenicity, the power of omics technologies is multiplied, resulting in a practical approach to novel biomarker development. This includes establishing and maintaining bioinformatics tools, data sources, networking platforms, and visualization platforms. The increasing availability of large-scale datasets and increased collaboration among scientists to share data will accelerate the understanding of mycobacterial networks to explain the complexity of replication, transcription, and translation during host-pathogen interactions and thus provide new insights into mycobacterium biology. Increased accessibility, combined with significant cost reductions for omics technologies and advancements in data analysis platforms, both for data management and convenience ensures that omics are common in biomarker, drug discovery, preclinical and clinical development. Due to the applied nature of omics approaches, omics technologies play a critical role in disease diagnosis and prevention. Omics methods have developed into valuable tools for discovering novel antimicrobial agents that are increasingly important to find. New omics are emerging to offer future biomarkers, and drug development and advancement of omics technologies can permit interpretations of metabolic rearrangements during infection and provide a more reliable understanding of pathogen physiology *in-vivo.* New omics techniques may open up unique opportunities for biological discovery and target identification as methodologies develop to improve sensitivity and overcome complexity.

## Author Contributions

NA: Conceptualization, writing original draft, review, & editing. SG: Writing original draft and review. DP: Writing original draft and review. All authors contributed to the article and approved the submitted version.

## Funding

This manuscript received no specific grant from any funding agency in the public, commercial, or not-for-profit sectors.

## Conflict of Interest

The authors declare that the research was conducted in the absence of any commercial or financial relationships that could be construed as a potential conflict of interest.

## Publisher’s Note

All claims expressed in this article are solely those of the authors and do not necessarily represent those of their affiliated organizations, or those of the publisher, the editors and the reviewers. Any product that may be evaluated in this article, or claim that may be made by its manufacturer, is not guaranteed or endorsed by the publisher.
